# Recombinant Immunomodulating Lentogenic or Mesogenic Oncolytic Newcastle Disease Virus for Treatment of Pancreatic Adenocarcinoma

**DOI:** 10.3390/v7062756

**Published:** 2015-06-11

**Authors:** Pascal Buijs, Stefan van Nieuwkoop, Vincent Vaes, Ron Fouchier, Casper van Eijck, Bernadette van den Hoogen

**Affiliations:** 1 Department of Surgery, Erasmus MC, University Medical Center Rotterdam, 's-Gravendijkwal 230, 3015 CE Rotterdam, The Netherlands; E-Mails: p.buijs@erasmusmc.nl (P.B.); c.vaneijck@erasmusmc.nl (C.E.); 2 Department of Viroscience, Erasmus MC, University Medical Center Rotterdam, 's-Gravendijkwal 230, 3015 CE Rotterdam, The Netherlands; E-Mails: s.vannieuwkoop@erasmusmc.nl (S.N.); v.vaes@erasmusmc.nl (V.V.); r.fouchier@erasmusmc.nl (R.F.)

**Keywords:** oncolytic virus, oncolytic virotherapy, Newcastle disease virus, pancreatic adenocarcinoma, innate immunity, immunotherapy

## Abstract

Oncolytic Newcastle disease virus (NDV) might be a promising new therapeutic agent for the treatment of pancreatic cancer. We evaluated recombinant NDVs (rNDVs) expressing interferon (rNDV-hIFNβ-F_0_) or an IFN antagonistic protein (rNDV-NS1-F_0_), as well as rNDV with increased virulence (rNDV-F_3aa_) for oncolytic efficacy in human pancreatic adenocarcinoma cells. Expression of additional proteins did not hamper virus replication or cytotoxic effects on itself. However, expression of interferon, but not NS1, resulted in loss of multicycle replication. Conversely, increasing the virulence (rNDV-F_3aa_) resulted in enhanced replication of the virus. Type I interferon was produced in high amounts by all tumor cells inoculated with rNDV-hIFNβ-F_0_, while inoculation with rNDV-NS1-F_0_ resulted in a complete block of interferon production in most cells. Inoculation of human pancreatic adenocarcinoma cells with rNDV-F_3aa_ caused markedly more cytotoxicity compared to rNDV-F_0_, while inoculation with rNDV-hIFNβ-F_0_ and rNDV-NS1-F_0_ induced cytotoxic effects comparable to those induced by the parental rNDV-F_0_. Evaluation *in vivo* using mice bearing subcutaneous pancreatic cancer xenografts revealed that only intratumoral injection with rNDV-F_3aa_ resulted in regression of tumors. We conclude that although lentogenic rNDVs harboring proteins that modulate the type I interferon pathway proteins do have an oncolytic effect, a more virulent mesogenic rNDV might be needed to improve oncolytic efficacy.

## 1. Introduction

Patients with pancreatic adenocarcinoma still have very poor survival rates, and current therapies are of limited effect [1,2]. Oncolytic Newcastle disease virus (NDV) could be a promising new therapeutic agent for the treatment of pancreatic cancer. Newcastle disease virus (NDV) has been described as a naturally occurring oncolytic virus as early as 1952 [3,4,5]. Since then, numerous clinical trials have employed wild type NDV strains either as a direct oncolytic agent, or as an oncolysate vaccine for treatment of patients with various types of advanced stage cancer [6,7,8,9,10,11]. Results of these early trials have been relatively disappointing, illustrated by the lack of further development of these treatment strategies. With the advent of recombinant DNA techniques it has become possible to genetically engineer NDV [12], and interest in the use of recombinant NDV (rNDV) as an oncolytic virus has revived over the last decade [13]. 

NDV selectively replicates in and destroys tumor cells while sparing normal cells, presumably because of defective interferon signaling pathways of the innate immune system in tumor cells. Previously, we reported that a lentogenic wild type NDV strain replicated in and was cytotoxic for 11 human pancreatic adenocarcinoma cell lines (HPACs) with high variability. These differences in the response of HPACs were not due to defects in innate immunity pathways as a number of these cell lines produced type I interferon (IFN) upon NDV infection [14]. We, and others, have also shown that IFN produced by tumor cells and the normal cells surrounding the tumor cells exerts anti-proliferative, pro-apoptotic and pro-inflammatory effects in tumor cells, possibly attributing to oncolytic efficacy [15,16,17,18,19]. Therefore, rNDVs armed with the IFN gene might result in a virus with higher oncolytic efficacy. However, the antiviral activity of IFN hampers NDV replication in tumor cells [14]. Viruses use different strategies to counteract the IFN pathway to increase infectivity and replication efficiency. The non-structural NS1 protein of influenza A virus is one of the most potent antagonists of the IFN response of the innate immunity characterized to date [20,21]. Oncolyis of tumor cells depend on efficient virus infection and replication, therefore, incorporation of an IFN antagonist, such as the NS1 protein of Influenza A in the genome of rNDV, could be a way to improve the oncolytic efficacy of rNDV. 

NDV strains are categorized in three different groups based on the severity of the disease they cause in birds: lentogenic, mesogenic, and velogenic and this classification correlates with their oncolytic properties in cancer cells. Increasing the virulence of rNDV has been shown to improve direct oncolytic efficacy most, resulting in preclinical studies using mesogenic virulent rNDVs expressing transgenes, such as IFN and the NS1 protein of influenza virus [13]. However, virulent NDV strains pose an environmental risk, as birds (specifically poultry) are very susceptible to infection with mesogenic or velogenic strains.

We hypothesize that arming lentogenic rNDV with IFN-modulating genes, either accelerating or blocking the IFN response, would improve the oncolytic effect of NDV sufficiently to circumvent using virulent rNDV with the associated potential biosafety risks. In the present study we compared lentogenic rNDVs expressing either hIFNβ or the IFN antagonistic protein (NS1) of the Influenza virus with those of a virulent rNDV (rNDV-F_3aa_) for direct oncolytic efficacy.

## 2. Materials and Methods

### 2.1. pNDV Cloning

A full-length cDNA clone of lentogenic NDV strain La Sota (pNDV-F_0_) and expression plasmids pCIneo-NP, pCIneo-P and pCIneo-L, as well as cloning plasmid pGEM-T-PM-cassette were kindly provided by Ben Peeters from the Central Veterinary Institute of Wageningen UR, The Netherlands [12,22]. To create a full length rNDV-F_0_ expressing either GFP, hIFN-β or NS1 protein of influenza strain A/PuertoRico/8/1934, a DNA fragment containing the open reading frame (ORF) encoding these proteins was inserted into the intergenic region between the P and M genes of pNDV-F_0_ flanked by appropriate NDV-specific transcriptional gene-start and gene-end signals. Cloning strategies resulted in full length NDV genomes that complied with the rule-of-six [23]. To create a full length NDV cDNA clone with a multibasic cleavage site in the fusion protein, the amino acid sequence of the protease cleavage site was changed from ^112^GRQGR↓L^117^ (lentogenic) to ^112^RRQRR↓F^117^ (mesogenic; pNDV-F_3aa_) by means of site-directed mutagenesis as described earlier [24]. Full-length NDV plasmids were sequenced using a 3130*xL* Genetic Analyzer (Life Technologies, Bleiswijk, The Netherlands) to exclude incidental mutations that could arise during the cloning process.

### 2.2. Recombinant Virus Rescue

Recombinant NDVs were rescued using a method adapted from the original method described previously [12]. Briefly, BSR-T7 cells were transfected with 5 µg full length pNDV, 2.5 µg pCIneo-NP, 1.25 µg pCIneo-P and 1.25 µg pCIneo-L using 10 µL lipofectamine (Life Technologies). Three days later, 200 µL BSR-T7 supernatant was injected into the allantoic cavity of 10 day old specified pathogen free (SPF) embryonated chicken eggs. After incubation in a humidified egg incubator at 37 °C for two or three days (mesogenic or lentogenic rNDVs, respectively), allantoic fluid was harvested and presence of virus demonstrated by hemagglutination assay [25]. Samples displaying hemagglutination were passaged once more in eggs to increase virus titer, and allantoic fluid was harvested after 2 or 3 days. Pooled fresh allantoic fluid was purified and concentrated by ultracentrifugation at 27.000 rpm for two h at 4 °C using a 30%/60% sucrose gradient. rNDV stocks were stored at −80 °C.

Recombinant influenza virus A/PuertoRico/8/34 was rescued and titrated as described before [26].

### 2.3. Titration of rNDV

Virus stocks were titrated by end point dilution assay in Vero-118 cells, as described before [14]. All infection experiments were performed in the presence of reduced concentration FBS HyClone (3%; Thermo Fischer Scientific, Breda, The Netherlands), without the addition of exogenous trypsin.

### 2.4. Characterization of rNDV

RNA from virus stocks was isolated using the High Pure RNA Isolation kit (Roche, Woerden, The Netherlands) following the manufacturer’s instructions and reverse transcribed into cDNA using a two-step protocol, as described before [14]. Amplicons generated with RT-PCR using primers flanking the P-M intergenic or F protein cleavage site region were sequenced to confirm the sequence of the inserted gene between the P and M genes and the protease cleavage site of the fusion protein (F_0_/F_3aa_).

Expression of NS1 protein was assayed in Vero-118 cells (mock-)inoculated at m.o.i. 3, with either rNDV-F_0_, rNDV-NS1-F_0_ or positive control influenza A/PuertoRico/8/34. Whole cell-lysates taken 18 h post-inoculation were subjected to sodium dodecyl sulfate-polyacrylamide gel electrophoresis (SDS-PAGE) and transferred to a Hybond-C Extra nitrocellulose membrane (GE Healthcare Life Sciences, Diegem, Belgium). Membranes were stained for NS1 using primary monoclonal mouse anti-influenza A NS1 antibody (AB_2011757/sc-130568; 1:1000 dilution; Santa Cruz Biotechnology, Heidelberg, Germany) and secondary peroxidase-conjugated polyclonal goat anti-mouse antibody (P0447; 1:2000 dilution; Dako, Heverlee, Belgium) or for β-tubulin using peroxidase-conjugated monoclonal rabbit anti-β-tubulin antibody (9099; 1:1000 dilution; Cell Signaling Technology, Leiden, The Netherlands). Chemiluminescent signals were generated using Amersham ECL Prime Western Blotting Detection Reagent (GE Healthcare Life Sciences) following manufacturer’s instructions and detected using a ChemiDoc MP system (Bio-Rad, Veenendaal, The Netherlands).

### 2.5. Cell Lines and Culture Conditions

The HPACs SU.86.86, HPAF-II, BxPC-3, PANC-1, MIA PaCa-2, Hs 700T, CFPAC, Hs 766T, AsPC-1, and Capan-2 were obtained from the American Type Culture Collection and authenticated using Short Tandem Repeat profiling [27]. Cells were not used more than 25 passages after thawing. HPACs, non-neoplastic human lung fibroblasts MRC-5, Vero-118, BSR-T7, and 293T cells were cultured as described before [14,28].

### 2.6. Replication Curves

For MIA PaCa-2 cells 3.0 × 10^6^ and for SU.86.86, HPAF-II, BxPC-3, PANC-1 and Hs 700T 1.5 × 10^6^ cells in T25 flasks (Corning, Amsterdam, The Netherlands) were inoculated at m.o.i. 0.1, in triplicate After a 1 h incubation, cells were washed three times with PBS and fresh medium was added. At time points 0, 2 12, 24, 48, and 96 h after washing, duplicates of 100 μL supernatant were collected, mixed with 100 μL 50% (*w/v*) sucrose, and frozen at −80 °C. Samples were titrated by end point dilution assay in quadruplicate as described before [14]. 

### 2.7. RNA Isolation and Quantitative Real-Time Polymerase Chain Reaction (qRT-PCR) for hIFNβ mRNA

Twenty-four hours after inoculation with rNDV at m.o.i. 3, cells were lysed with 300 µL lysis buffer of the Total Nucleic Acid Isolation kit (Roche) and RNA was isolated using a MagNA Pure LC machine (Roche) following the manufacturer’s instructions. qRT-PCR (30 cycles) was performed with 20 μL RNA in an ABI PRISM 7000 Sequence Detection System (Life Technologies), using TaqMan gene expression assay for human IFNβ1 (Hs00277188_s1, Life Technologies). The primers in this assay map to the extreme 3′ end of the hIFNβ gene (Hs00277188_s1, www.lifetechnologies.com) [29], with the reverse primer annealing downstream of the stop codon of the hIFNβ coding sequence. This region is not present in the rNDV-hIFNβ-F_0_ virus. Therefore, the qRT-PCR assay is not able to detect IFN-mRNA transcribed from the virus, and the assay will only detect endogenous transcribed IFN-mRNA. To detect both endogenous and exogenous expressed IFN, primers and probes mapping in the IFN coding region were used with an in-house developed assay and β-actin was used as household gene). The sequences of the primers and probes for the IFN-coding region and β-actin have been described before [30]. Results are presented as fold change of inoculated samples versus mock-inoculated samples (duplicates), calculated using the 2^−ΔΔ^^*C*^_T_ method [31].

### 2.8. IFN Measurement with Luciferase Bioassay

Cells were inoculated in triplicate at m.o.i 3 and 24 h post inoculation, supernatants were collected and assayed for IFN contents using a bioassay as described before [14]. IFN produced by NDV-inoculated cells is presented as fold change in luminescence compared to mock inoculated cells.

### 2.9. Cytotoxicity Assay

Quadruplicates of 2 × 10^4^ cells per well in 96-well plates (Corning) were either mock inoculated or inoculated with rNDV at different m.o.i. (range 0.0001–100). After 48 h, 100 μL fresh medium was added. At time point 120 h post inoculation, cell viability was determined using the CytoTox 96 Non-Radioactive Cytotoxicity Assay (Promega, Leiden, The Netherlands), as described before [14]. Prism for Windows version 5.03 (GraphPad software, La Jolla, CA, USA) was used to analyze data using the log(inhibitor) vs. normalized response (variable slope) function to obtain LD_50_ values. The extra sum-of-squares F test was used to compare LD_50_ values. *p*-values of <0.05 were considered statistically significant. 

### 2.10. Ethics Statement

All experiments involving animals were conducted strictly according to European guidelines (EU directive on animal testing 86/609/EEC) and Dutch legislation (Experiments on Animals Act, 1997). The experimental protocol was reviewed and approved by an independent animal experimentation ethical review committee, not affiliated with Erasmus MC (DEC consult number EMC2921).

### 2.11. Animals and Experimental Design

Groups of 30 athymic nude mice (strain NMRI-*Foxn1^nu^*; Charles River, Sulzfeld, Germany) were injected subcutaneously in their flank with 3 × 10^6^ SU.86.86, BxPC-3 or MIA PaCa-2 cells. Tumor width (w) and length (l) were measured using a digital caliper (VWR International, Amsterdam, The Netherlands) and tumor volume was calculated using the modified ellipsoid formula w^2^ × l/2 [32,33]. Tumors were allowed to grow until the average tumor volume per group reached 50 mm^3^ (3–5 weeks) and animals were appointed randomly stratified for tumor size to one of five treatment groups: PBS, rNDV-F_0_, rNDV-hIFNβ-F_0_, rNDV-NS1-F_0_ or rNDV-F_3aa_. Animals were injected intratumorally every other day for a total of 4 injections with 5 × 10^7^ TCID_50_ rNDV in a total volume of 50 µL or an equivalent volume PBS. After injection, tumor sizes were recorded two times weekly. Animals were euthanized if tumor volume exceeded 2000 mm^3^, non-healing tumor ulcerations or excessive weight loss occurred, and ultimately 40 days after first injection with rNDV or PBS. Last observed tumor volumes were carried forward to calculate median volumes per group. Continuous data were compared between the groups using the Mann–Whitney U-test. One-sided *p*-values of <0.05 were considered statistically significant.

## 3. Results

### 3.1. Cloning, Rescue and Characterization of Recombinant NDVs

**Figure 1:**
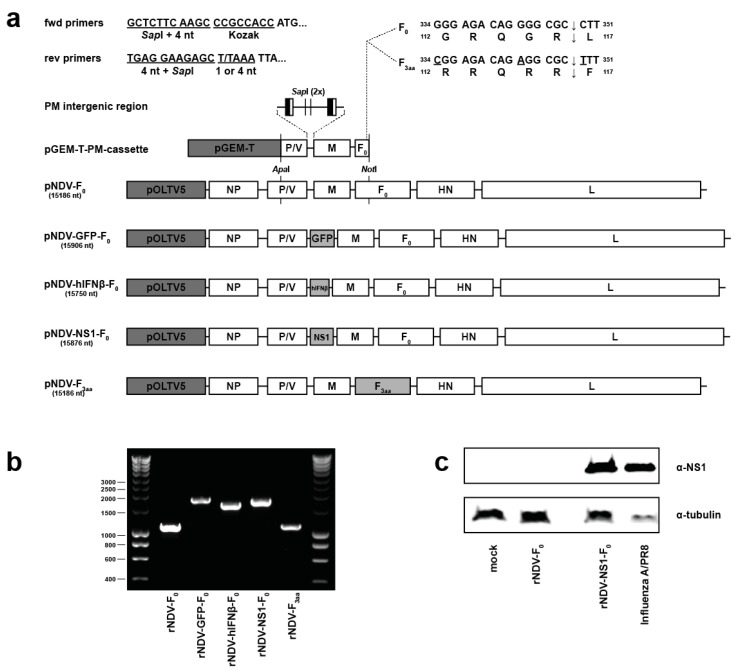
Generation and characterization of rNDVs. (**a**) Cloning strategy to obtain full length NDV plasmids. The nucleotide length of the NDV genome is noted below the plasmid names and all full-length plasmids and pGEM-T-PM-cassette are drawn to scale. The 3′-terminal leader, intergenic regions and 5′-terminal trailer are shown as horizontal lines. Gene-end and gene-start sequences in the PM intergenic region are depicted as vertical black and white rectangles, respectively. fwd: forward; rev: reverse; nt: nucleotides; pGEM-T: plasmid backbone of pGEM-T-PM-cassette; NP: nucleoprotein gene; P: phosphoprotein gene; V: accessory V gene; M: matrix gene; F: fusion gene; HN: hemagglutinin-neuraminidase gene; L: large protein gene; (**b**) PCR product of PM intergenic region. RNA of indicated rNDV stocks was reverse transcribed into cDNA and the PM intergenic region was amplified with RT-PCR using flanking primers; (**c**) Western blot for expression of NS1 protein. Whole cell lysates of Vero-118 cells (mock-) inoculated with indicated viruses were assayed for expression of the NS1 protein or tubulin protein.

Five different rNDVs were generated which were used throughout this study: rNDV-F_0_, rNDV-GFP-F_0_, rNDV-hIFNβ-F_0_, rNDV-NS1-F_0_ and rNDV-F_3aa_ (Figure 1a). Amplicons of the P-M intergenic (Figure 1b) and F protein cleavage site were sequenced to confirm identity of the rNDV stocks. Upon titration of virus stocks, only infection with rNDV-F_3aa_ lead to syncytia formation in cell cultures, characteristic for infection with mesogenic NDV (data not shown). In addition, Western blot assay confirmed the expression of NS1 by rNDV-NS1-F_0_ (Figure 1c).

### 3.2. Replication Kinetics of rNDVs

To test whether the expression of additional transgenes or a change in cleavability of the F protein had an effect on replication kinetics of rNDVs, replication curves were generated for the different viruses on six different HPACs. HPACs were selected based on replication efficiency of the wild type virus as reported in our previous study [14].

**Figure 2:**
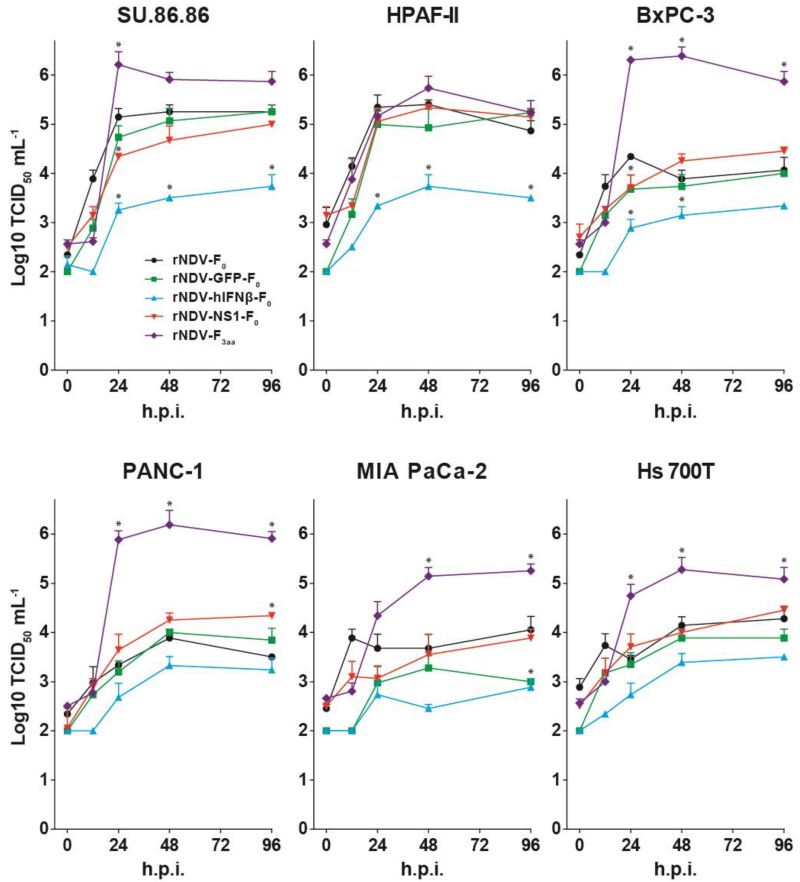
Replication kinetics of rNDVs in 6 different human pancreatic adenocarcinoma cell lines (HPACs). Cells were inoculated in triplo with m.o.i. 0.1 and samples were taken at indicated time points and titrated by end-point dilution assay in Vero-118 cells. Means and standard deviations of duplicate titrations are plotted. H.p.i.: hours post inoculation. * = *p* < 0.05 *vs*. rNDV-F_0_ (one-way ANOVA + Bonferroni post-test), tested for time points 24, 48 and 96 h.p.i.

Upon inoculation, no significant differences were observed for replication of the lentogenic viruses rNDV-F_0,_ rNDV-GFP-F_0_, and rNDV-NS1-F_0_ on all cellsSU.86.86 and PHPAF-II cells supported replication of these viruses to high titers, while replication was less efficient in the other four cell lines (Figure 2).

Inoculation with rNDV-hIFNβ-F_0_ resulted in attenuated replication in all cells, with significant lower titers in SU.86.86, HPAF-II and BxPC-3 cells compared to inoculation with rNDV-F_0_. 

In contrast, inoculation with the mesogenic virus rNDV-F_3aa_ resulted in efficient multicycle replication in most cell lines. Inoculation of SU.86.86 with rNDV-F_3aa_ resulted in significant higher titers at *t* = 24 compared to those of rNDV-F_0_ and after this time point titers of rDNV-F_3aa_ declined, due to the loss of cells as an effect of the efficient replication. Inoculation with rNDV-F_3aa_ of BxPC-3, Panc-1, MIA PaCa-2, and Hs 700T resulted in significant higher titers at *t* = 24, 48 and 96 compared to those of rDNV-F_0_. Although the titers for rNDV-F3aa in HPAF-II cells were higher than those of the other viruses in HPAF-II cells these differences were not significant (Figure 2).

### 3.3. Modulation of IFN Response by rNDVs

As IFN has a direct effect on oncolyis, the capacity of the viruses to induce, enhance or block IFN production was evaluated. Ten HPACs, as well as Vero cells (lacking the endogenous IFN genes) and MRC-5 cells (fully IFN competent), were inoculated with the five different rNDVs and at 24 h after inoculation both hIFNβ protein production as well as gene expression levels were measured. The gene expression assay for endogenously expressed IFN maps to the extreme 3′ end of the hIFNβ gene, with the reverse primer annealing downstream of the stop codon of the hIFNβ coding sequence. This region is not present in the rNDV-hIFNβ-F_0_ virus, thus only the endogenous transcribed IFN-mRNA is detected and not IFN-mRNA transcribed from the rNDV-hIFNβ-F_0_ virus.

**Figure 3:**
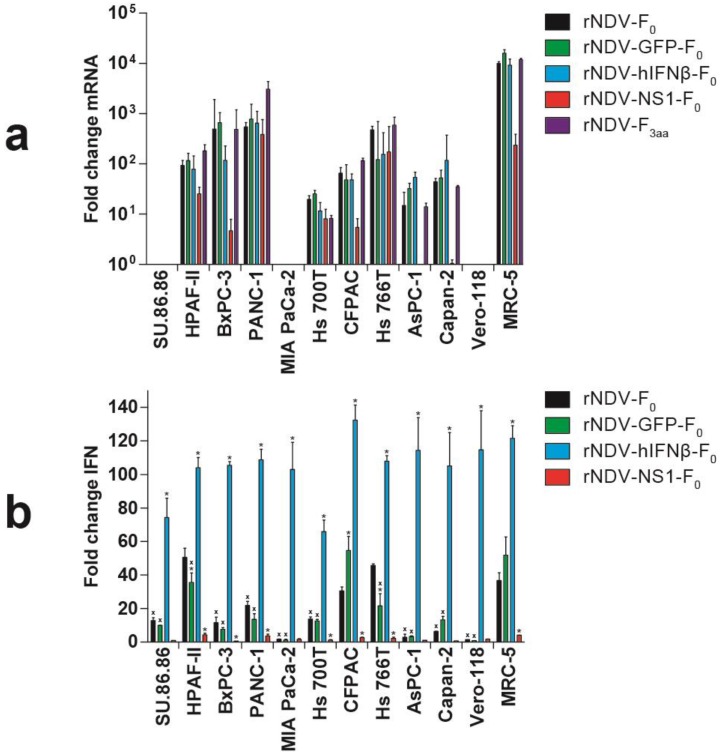
IFN responses upon inoculation with rNDVs. Cells were either mock inoculated or inoculated with the indicated rNDV at m.o.i. 3. After 24 h: (**a**) RNA was isolated and quantitative real-time polymerase chain reaction (qRT-PCR) was performed for endogenous hIFNβ mRNA. Results are presented as fold change gene induction of treated *versus* mock treated cells calculated using the 2^−ΔΔ^^*C*^_T_ method [31]. Means and ranges of triplicate experiments are plotted; (**b**) Supernatants were tested for functional IFN protein content using an ISRE-luc bioassay [14]. Results are presented as fold change in luminescence compared to mock inoculated cells. Means and standard deviations of triplicate experiments are plotted. x = *p* < 0.05 *vs.* MRC-5 (one-way ANOVA + Bonferroni post-test), * = *p* < 0.05 *vs*. rNDV-F_0_ (one-way ANOVA + Bonferroni post-test).

Inoculation with rNDV-F_0_ or rNDV-GFP-F_0_ resulted in upregulation of endogenous hIFNβ gene expression in MRC-5 cells and in eight of the HPACs. No upregulation was observed in in SU.86.86, MIA PaCa-2 and, as expected, Vero cells. These findings were mostly in agreement with our findings using wild type NDV, except for BxPC-3 cells [14]. Inoculation with rNDV-hIFNβ-F_0_ did not change this pattern of endogenous hIFNβ gene expression. An in-house assay detecting expression of both endogenous and exogenous IFN genes revealed similar expression levels as detected with the validated endogenous assay for cells inoculated with rNDV-F_0_ or rNDV-GFP-F_0_, but, as expected, higher expression levels for rNDV-hIFNβ-F_0_ inoculated cells (data not shown). With this assay, expression of exogenous expressed hIFNβ mRNA was also detected in rNDV-GFP-F_0_ inoculated SU86.86, Mia PaCa-2 and Vero cells, in which endogenous IFN genes are absent or not upregulated (Figure 3A). Inoculation with rNDV-NS1-F_0_ resulted in a marked decrease in endogenous hIFNβ gene expression levels in HPAF-II, BxPC-3, CFPAC, AsPC-1, Capan-2 cells, and MRC-5 fibroblasts, while no differences were observed in PANC-1 and Hs 766T cells, compared to expression levels in these cells inoculated with rNDV-F_0_. 

To test whether the differences in expression of the hIFNβ genes also resulted in differences in protein production, functional IFN protein content was determined in the supernatants of inoculated cells. Due to biosafety issues, we could not measure IFN in supernatants of cells inoculated with rNDV-F_3aa_. 

Upon inoculation with rNDV-F_0_, cells demonstrated variation in the extent of IFN production. Six of the 10 HPACs produced significant less IFN compared to IFN-competent MRC-5 cells, while HPAF-II, CFPAC, and Hs 766T had a similar range of IFN production. Incorporation of GFP in the genome of rNDV-F_0_ did not change this pattern in most HPACs. Only HPAF-II and Hs 766T cells produced significant less IFN upon inoculation with rNDV-GFP-F_0_ compared to inoculation with rNDV-F_0_, while the opposite was observed for CFPAC cells. 

Upon inoculation with rNDV-hIFNβ-F_0_, all cells produced significant higher amounts of IFN compared to inoculation with rNDV-F_0_, due to viral expression of the exogenous hIFNβ gene (Figure 3b). This exogenously expressed IFN did not induce upregulation of endogenous hIFNβ gene expression in SU.86.86, MIA PaCa-2 and, of course, Vero-118 cells (as seen in Figure 3a). Inoculation of HPACs and MRC-5 cells with rNDV-NS1-F_0_ resulted in a significant decrease of IFN production in all these cells as compared to cells inoculated with rNDV-F0 (Figure 3b).

### 3.4. In Vitro Cytotoxicity

**Figure 4:**
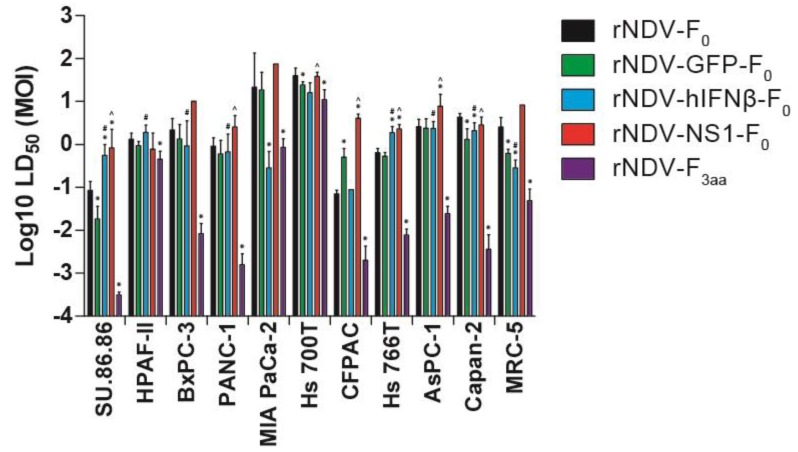
Median LD_50_ upon inoculation with rNDVs. Cells were either mock inoculated (not shown; set as 100% viable) or inoculated with rNDV at different m.o.i. (range 0.0001–100). Cytotoxicity was measured after 5 days by LDH assay and LD_50_ values were calculated. Means and ranges of LD_50_ calculations are plotted. * = *p* < 0.05 as compared rNDV-GFP-F_0_, rNDV-hIFNβ-F_0_ and rNDV-F_3aa_ compared to rNDV-F_0_. # = *p* < 0.05, rNDV-F_3aa_ compared to rNDV-hIFNβ-F_0_. ^ = *p* < 0.05 rNDV-F3_aa_ compared to rNDV-NS1-F_0_.

To evaluate the effect of increasing the virulence or incorporation of hIFNβ or NS1 in the genome of rNDV on direct oncolysis, HPACs and MRC-5 fibroblasts were inoculated with serial dilutions of the viruses and the median lethal dose (LD_50_) was determined for each rNDV-cell line combination. Mean LD_50_ levels for rNDV-F_0_ and rNDV-GFP-F_0_ were generally comparable between cell lines, although SU.86.86, HS 700T, Capan-2 and MRC-5 demonstrated significant lower cytotoxicity upon inoculation with rNDV-GFP-F_0_ compared to rNDV-F_0_, while CFPAC cells demonstrated higher cytotoxicity for rNDV-GFP-F_0_ compared to rNDV-F_0_ (* above green bars in Figure 4). This indicates that incorporation of inert transgenes might have some effect on oncolytic efficacy, but with similar variation in reaction between the different cells. 

Compared to rNDV-F_0_, expression of NS1 from rNDV-NS1-F_0_ did not improve the oncolytic effect for most cells. Equal LD_50_ values were detected for rNDV-F_0_ and rNDV-NS1-F_0_ for HPAF-II, Hs 700T, Hs 766T, and Capan-2 cells, with even higher LD_50_ values the other cells. Compared to rNDV-F_0_, expression of exogenous IFN from rNDV-hIFNβ-F_0_ decreased the oncolytic effect significantly in SU.86.86 cells and HS766T cells, but increased oncolysis significantly in Mia PaCa-2, Capan-2, and MRC-5 cells. Although statistical analyses revealed differences in the oncolytic effect between rNDV-GFP-F_0_, rDNV-NS1-F_0_ and rNDV-hIFNβ-F_0_, with variation in the response of the different cells, the results shown in Figure 4 demonstrate that inoculation with the more virulent rNDV-F_3aa_ results in a clear improvement of the oncolytic effect. 

Inoculation with rNDV-F_3aa_ resulted in a significant increase in oncolysis in all HPACs when compared to inoculation with rNDV-F_0_ (* above purple bars), for 7 out of 10 HPACs (SU.86.86, PANC-1, Hs 700T, CFPAC, Hs 766T, AsPC-1, Capan-2) when compared to rNDV-NS1-F_0_ (^ above red bars), and for 7 out of 10 HPACs (SU.86.86, HPAF, BxPC-3, PANC-1, Hs 766T, AsPC-1 and Capan-2) when compared to rNDV-hIFNβ-F_0_ (# above blue bars). Compared to rNDV-hIFNβ-F_0_, which revealed improved oncolysis in Mia PaCa-2 and Capan-2 cells, rNDV-F_3aa_ had significantly increased oncolytic effect on Capan-2 cells but an equal effect on Mia PaCa-2 cells. In general, HPAF-II, Mia PaCa-2 and Hs 700T were most resistant to the oncolytic effects induced by the five viruses, including the virulent rNDV-F_3aa_.

### 3.5. In Vivo Efficacy: rNDV-F_3aa_ Effective in Multiple Models

After having shown oncolytic and efficacy *in vitro*, we extended our evaluation of rNDVs to *in vivo* experiments in immune-deficient subcutaneous xenograft mouse models for pancreatic adenocarcinoma, using US.86.86, BxPC-3, and MIA PaCa-2 cells. Before starting treatment experiments, toxicity of all viruses was tested in small groups of mice (*n* = 3), using per group escalating doses starting from 1 × 10^6^ TCID_50_ up to four times 5 × 10^7^ TCID_50_ injected intravenously or subcutaneously. None of the injected mice showed adverse effects or excessive weight loss during these experiments, and we concluded it was safe to inject mice with the highest dose. Additionally, based on a separate pilot experiment, we decided to inject mice intratumorally with rNDV since this gave better treatment responses compared to intravenous injection.

**Figure 5:**
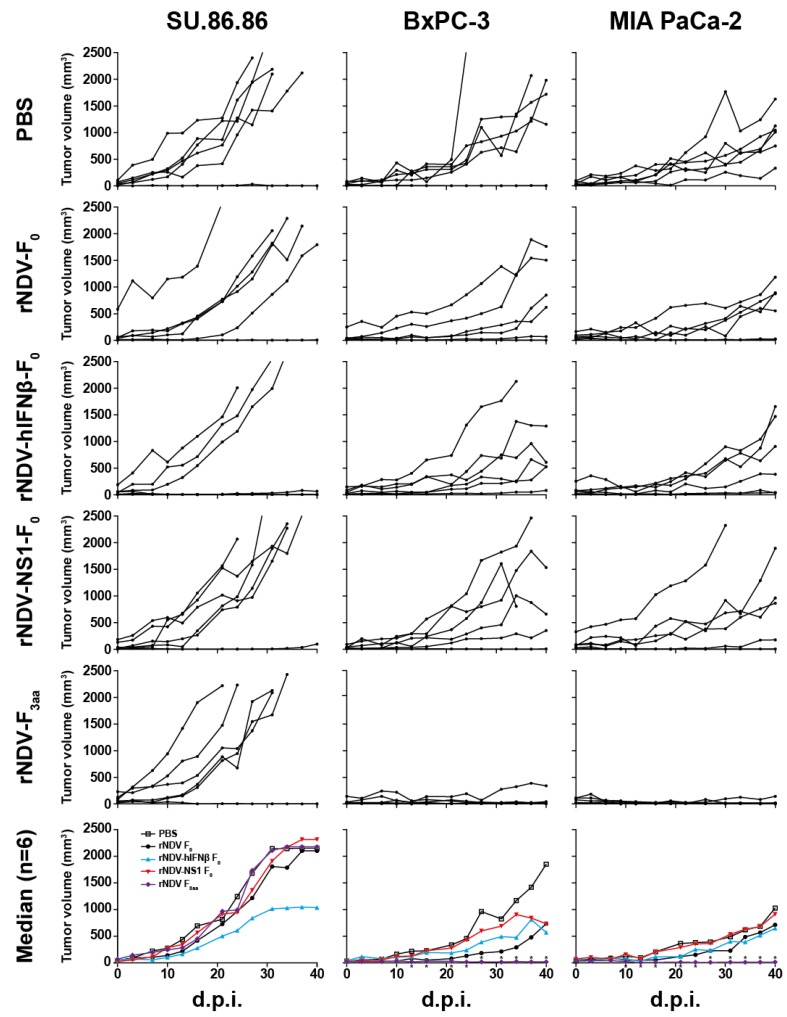
Efficacy of rNDV treatment in a mouse model using SU.86.86, BxPC-3 or MIA PaCa-2 subcutaneous tumor xenografts. Animals were treated and evaluated as described in the material and methods section. Graphs with specific xenograft-treatment combinations depict individual mouse tumor volumes with black lines and dots. Last observed tumor volumes were carried forward to calculate median volumes per group in the lowest plots. *: *p* < 0.05 as compared to PBS group. D.p.i.: days post first injection.

SU.86.86 tumor xenografts were resistant to all rNDV treatments, even to multiple injections with mesogenic rNDV-F_3aa_ (Figure 5). While BxPC-3 and MIA PaCa-2 tumor xenografts were resistant to injection with rNDV-F_0_, rNDV-hIFNβ-F_0_ and rNDV-NS1-F_0_, they did respond to injection with the mesogenic rNDV-F_3aa_. Treatment with rNDV-F_3aa_ resulted in tumor regression in five out of six animals and median tumor sizes were significantly smaller starting at day 13 and 10 after the first injection for BxPC-3 and MIA PaCa-2 tumor xenografts respectively (*p* < 0.05). Upon necropsy of these animals, only very small residual tumors were found, as opposed to the mostly large tumors in animals treated with either PBS or lentogenic rNDVs.

## 4. Discussion

In our efforts to further develop oncolytic viro-therapy for pancreatic cancer, we focused our attention on the use of recombinant NDVs. In our previous study, we evaluated the efficacy of a wild type lentogenic oncolytic NDV strain in a panel of 11 HPACs. This demonstrated a high degree of variation between the cells in their response to inoculation with NDV, not only in oncolysis but also in activation of the IFN response [14]. We hypothesized that increasing the oncolytic effects of NDV would overcome this variability. Various strategies have been reported to improve the efficacy of oncolytic rNDVs: transfer of therapeutic or immunomodulating transgenes [24,34,35,36,37], targeting of tumor cells with modified attachment proteins [38,39], and increasing virulence by increasing the cleavability of the F protein [24,35,40,41,42,43]. As increased virulence can raise biosafety issues, we aimed to improve the direct oncolytic effect of non-virulent (lentogenic) rNDVs by expressing IFN modulating genes and we compared their efficacy with a more virulent (mesogenic) rNDV.

Expression of a non-modulating transgene (GFP) or the NS1 protein of the Influenza virus from a lentogenic rNDV did not change the replication kinetics of rNDV-F_0_ in a set of six HPACs, but expression of high levels of exogenous hIFNβ from rNDV-hIFNβ-F_0_ hampered virus replication in susceptible HPACs. This was not surprising, since we showed earlier that HPACs have mostly intact IFN signaling pathways and replication of NDV is sensitive to IFN treatment [14]. In contrast to the lentogenic viruses, the mesogenic rNDV-F_3aa_ was capable of multicycle replication in all HPACs tested. This higher efficiency in replication correlated with the higher cytotoxic effects for NDV-F_3aa_ in most HPACs, indicating this to be the oncolytic rNDV with the highest oncolytic efficacy. This is in agreement with other studies that reported on the oncolytic efficacy of mesogenic rNDVs [24,35,40,41,42,43]. However, three HPACs (HPAF-II, MIA PaCa-2, and Hs 700T) remained somewhat resistant to rNDV-F_3aa_-induced cytotoxicity, and it remains to be determined why some cells are more resistant than others. Future experiments should elaborate more on the etiology of differences in susceptibility of HPACs for the oncolytic effect of NDV. These experiments should not only focus on innate immunity, but also on differences in apoptotic, necrotic, autophagy and/or immunogenic cell death pathways. Elucidating knowledge on the traits defining susceptibility to NDV induced oncolytic effects would allow improvement of oncolytic NDV to also attack relatively resistant tumor cells. As previously reported for wild type NDV, inoculation with rNDV-F_0_ resulted in IFN production by a number of HPACs, but this did not always correlate with virus replication kinetics or oncolytic effects induced by rNDV-F_0_ [14]. Inoculation with rNDV-NS1-F_0_ resulted in markedly reduced hIFNβ gene expression levels in most HPACs capable of expressing endogenous hIFNβ and, more interestingly, in almost complete absence of IFN production in almost all HPACs. Indeed, it is known that NS1 of influenza A is a potent blocker of IFN induction by, among other mechanisms, suppressing RIG-I receptor signaling, IRF3 dimerization and subsequent IFNβ promoter activation [20,21]. Expression of exogenous hIFNβ from rNDV-hIFNβ-F_0_ did not lead to increased expression levels of the endogenous hIFNβ gene, but did result in production of high amounts of hIFNβ protein. This illustrates that lentogenic rNDV-F_0_ is very suitable as a (transient) gene therapy vector, as the (additional) production of IFN can only be attributed to viral hIFNβ gene expression. Interestingly, the lower replication of rNDV-hIFNβ-F_0_ did not always result in significant lower cytotoxicity in most HPACs (such as Mia Paca-2 cells), indicating that hIFNβ might have a cytotoxic effect by itself. A recent publication demonstrated that most HPACs are susceptible to exogenous hIFNβ treatment, but SU.86.86 cells were found to be relatively resistant [18]. In line with these findings, inoculation of SU.86.86 cells with rNDV-hIFNβ-F_0_ lead to lower cytotoxicity as compared to inoculation with rNDV-F_0_. Apparently, rNDV-hIFNβ-F_0_ replication and resulting cytotoxicity is hampered by the exogenous hIFNβ produced by inoculated SU.86.86 cells, while SU.86.86 cells are insensitive to the cytotoxic effects of hIFNβ. These findings are also in line with studies showing that incorporation of IFNβ genes into oncolytic viruses such as vesicular stomatitis virus or vaccinia virus leads to lower virus replication in cell lines with intact IFN signaling pathways [44,45,46,47], and, previously, we showed that most HPACs do have intact and functional innate immune pathways [14].

We observed up regulation of hIFNβ mRNA and functional IFN protein production in BxPC-3 cells, which was in conflict with the results of our previous study [14]. Other groups have also noted an unstable phenotype of BxPC-3 [48] and the difference between our findings in this study and the previously published one may be attributed to higher passage numbers of BxPC-3 cells used earlier.

Exogenous expression of influenza NS1 protein from rNDV-NS1-F_0_ did not change replication kinetics in the inoculated HPACs. However, it did lead to less cytotoxicity in most HPACs, indicating again that IFN has a cytotoxic effect on these cells. This is in contrast with results reported for a mesogenic rNDV expressing NS1 (rNDV-NS1-F_3aa_), which induced enhanced tumor cell killing due to inhibition of apoptosis, leading to increased syncytia formation [34]. 

*In vivo* experiments using a subcutaneous xenograft tumor model using PxPC-3 or MIA Paca-2 cells in immune-deficient mice demonstrated that intratumoral treatment with mesogenic rNDV-F_3aa_ induced tumor regression or stabilization. In contrast, SU.86.86 tumors, which are highly susceptible for oncolytic rNDV-F_3aa_ treatment *in vitro*, did not respond to injection with rNDV-F_3aa_ *in vivo*. SU.86.86 tumors showed a relatively aggressive growth rate in this mouse model when compared to the other tumor bearing groups, which might explain why rNDV-F_3aa_ treatment was not successful in achieving tumor regression or stabilization. These results illustrate again that variation in response and heterogeneity of tumors contributes to the efficacy of oncolytic viro-therapy. Intratumoral treatment with the lentogenic viruses did not lead to direct oncolytic effects in this model, indicating that, for direct oncolytic effects, the mesogenic rNDV-F_3aa_ is most effective.

The immune system is thought to contribute to the efficacy of oncolytic viruses and to efficient clearance of the virus from healthy cells [19]. In this study we have focused on the direct oncolytic effects of the virus and the contribution of IFN to this, in absence of the immune system. It might well be possible that the transgenes expressed by the lentogenic viruses would increase the efficacy in an immune competent model, however based on our results we expect a higher efficacy of rNDV-F_3aa_ in these models. Evaluation of the beneficial effects of the immune system to the oncolytic effects of rNDV-F_3aa_ needs to be performed in an immune-competent model for pancreatic tumors. Although transgenic animal models that mimic the natural development of pancreatic tumors have been created, at the moment these are limited and difficult to employ for evaluation of the efficacy of oncolytic viruses. 

In conclusion, expression of exogenous IFN modulating genes from lentogenic rNDVs does not significantly enhance direct oncolyis induced by these viruses compared to those induced by a more virulent virus. However, increasing the virulence of rNDV by increasing cleavability of the F protein lead to a significant improvement of oncolytic activity of recombinant NDV. For further development of virulent rNDV for oncolytic viro-therapy, the biosafety risks of the virus for birds and poultry should be addressed. In addition, knowledge should be elucidated on the heterogeneity of pancreatic tumors and on the traits defining susceptibility to NDV induced oncolytic effects.

## References

[1] Sultana A., Smith C.T., Cunningham D., Starling N., Neoptolemos J.P., Ghaneh P. (2007). Meta-analyses of chemotherapy for locally advanced and metastatic pancreatic cancer. J. Clin. Oncol..

[2] Neoptolemos J.P., Stocken D.D., Bassi C., Ghaneh P., Cunningham D., Goldstein D., Padbury R., Moore M.J., Gallinger S., Mariette C. (2010). Adjuvant chemotherapy with fluorouracil plus folinic acid vs gemcitabine following pancreatic cancer resection: A randomized controlled trial. JAMA.

[3] Moore A.E., Diamond L.C., Mackay H.H., Sabachewsky L. (1952). Influence of hemagglutinating viruses on tumor cell suspensions. Ii. Newcastle disease virus and ehrlich carcinoma. Proc. Soc. Exp. Biol. Med..

[4] Prince A.M., Ginsberg H.S. (1957). Studies on the cytotoxic effect of newcastle disease virus (NDV) on ehrlich ascites tumor cells. Ii. The mechanism and significance of in vitro recovery from the effect of ndv. J. Immunol..

[5] Prince A.M., Ginsberg H.S. (1957). Studies on the cytotoxic effect of newcastle disease virus (NDV) on ehrlich ascites tumor cells. I. Characteristics of the virus-cell interaction. J. Immunol..

[6] Lorence R.M., Roberts M.S., O'Neil J.D., Groene W.S., Miller J.A., Mueller S.N., Bamat M.K. (2007). Phase 1 clinical experience using intravenous administration of pv701, an oncolytic newcastle disease virus. Curr. Cancer Drug Targets.

[7] Freeman A.I., Zakay-Rones Z., Gomori J.M., Linetsky E., Rasooly L., Greenbaum E., Rozenman-Yair S., Panet A., Libson E., Irving C.S. (2006). Phase I/II trial of intravenous NDV-HUJ oncolytic virus in recurrent glioblastoma multiforme. Mol. Ther..

[8] Pecora A.L., Rizvi N., Cohen G.I., Meropol N.J., Sterman D., Marshall J.L., Goldberg S., Gross P., O'Neil J.D., Groene W.S. (2002). Phase i trial of intravenous administration of pv701, an oncolytic virus, in patients with advanced solid cancers. J. Clin. Oncol..

[9] Schirrmacher V. (2005). Clinical trials of antitumor vaccination with an autologous tumor cell vaccine modified by virus infection: Improvement of patient survival based on improved antitumor immune memory. Cancer Immunol. Immunother..

[10] Cassel W.A., Murray D.R. (1992). A ten-year follow-up on stage ii malignant melanoma patients treated postsurgically with newcastle disease virus oncolysate. Med. Oncol. Tumor Pharmacother..

[11] Sinkovics J.G., Horvath J.C. (2000). Newcastle disease virus (ndv): Brief history of its oncolytic strains. J. Clin. Virol..

[12] Peeters B.P., de Leeuw O.S., Koch G., Gielkens A.L. (1999). Rescue of newcastle disease virus from cloned cdna: Evidence that cleavability of the fusion protein is a major determinant for virulence. J. Virol..

[13] Zamarin D., Palese P. (2012). Oncolytic newcastle disease virus for cancer therapy: Old challenges and new directions. Future Microbiol..

[14] Buijs P.R., van Eijck C.H., Hofland L.J., Fouchier R.A., van den Hoogen B.G. (2014). Different responses of human pancreatic adenocarcinoma cell lines to oncolytic newcastle disease virus infection. Cancer Gene Ther..

[15] Morak M.J., van Koetsveld P.M., Kanaar R., Hofland L.J., van Eijck C.H. (2011). Type I interferons as radiosensitisers for pancreatic cancer. Eur. J. Cancer.

[16] Vitale G., van Eijck C.H., van Koetsveld Ing P.M., Erdmann J.I., Speel E.J., van der Wansem Ing K., Mooij D.M., Colao A., Lombardi G., Croze E. (2007). Type i interferons in the treatment of pancreatic cancer: Mechanisms of action and role of related receptors. Ann. Surg..

[17] Levy D.E., Garcia-Sastre A. (2001). The virus battles: Ifn induction of the antiviral state and mechanisms of viral evasion. Cytokine Growth Factor Rev..

[18] Booy S., van Eijck C.H., Dogan F., van Koetsveld P.M., Hofland L.J. (2014). Influence of type-i interferon receptor expression level on the response to type-I interferons in human pancreatic cancer cells. J. Cell. Mol. Med..

[19] Zamarin D., Holmgaard R.B., Subudhi S.K., Park J.S., Mansour M., Palese P., Merghoub T., Wolchok J.D., Allison J.P. (2014). Localized oncolytic virotherapy overcomes systemic tumor resistance to immune checkpoint blockade immunotherapy. Sci. Transl. Med..

[20] Van de Sandt C.E., Kreijtz J.H., Rimmelzwaan G.F. (2012). Evasion of influenza a viruses from innate and adaptive immune responses. Viruses.

[21] Kochs G., Garcia-Sastre A., Martinez-Sobrido L. (2007). Multiple anti-interferon actions of the influenza a virus ns1 protein. J. Virol..

[22] Kortekaas J., Dekker A., de Boer S.M., Weerdmeester K., Vloet R.P., de Wit A.A., Peeters B.P., Moormann R.J. (2010). Intramuscular inoculation of calves with an experimental newcastle disease virus-based vector vaccine elicits neutralizing antibodies against rift valley fever virus. Vaccine.

[23] Peeters B.P., Gruijthuijsen Y.K., de Leeuw O.S., Gielkens A.L. (2000). Genome replication of newcastle disease virus: Involvement of the rule-of-six. Arch. Virol..

[24] Vigil A., Park M.S., Martinez O., Chua M.A., Xiao S., Cros J.F., Martinez-Sobrido L., Woo S.L., Garcia-Sastre A. (2007). Use of reverse genetics to enhance the oncolytic properties of newcastle disease virus. Cancer Res..

[25] Hirst G.K. (1942). The quantitative determination of influenza virus and antibodies by means of red cell agglutination. J. Exp. Med..

[26] De Wit E., Spronken M.I., Bestebroer T.M., Rimmelzwaan G.F., Osterhaus A.D., Fouchier R.A. (2004). Efficient generation and growth of influenza virus a/pr/8/34 from eight cdna fragments. Virus Res..

[27] Masters J.R., Thomson J.A., Daly-Burns B., Reid Y.A., Dirks W.G., Packer P., Toji L.H., Ohno T., Tanabe H., Arlett C.F. (2001). Short tandem repeat profiling provides an international reference standard for human cell lines. Proc. Natl. Acad. Sci. USA.

[28] Herfst S., de Graaf M., Schickli J.H., Tang R.S., Kaur J., Yang C.F., Spaete R.R., Haller A.A., van den Hoogen B.G., Osterhaus A.D. (2004). Recovery of human metapneumovirus genetic lineages a and b from cloned cDNA. J. Virol..

[29] Hs00277188_s1. http://www.lifetechnologies.com/order/genome-database/details/gene-expression/Hs00277188_s1.

[30] Spann K.M., Tran K.C., Chi B., Rabin R.L., Collins P.L. (2004). Suppression of the induction of alpha, beta, and lambda interferons by the ns1 and ns2 proteins of human respiratory syncytial virus in human epithelial cells and macrophages [corrected]. J. Virol..

[31] Livak K.J., Schmittgen T.D. (2001). Analysis of relative gene expression data using real-time quantitative PCR and the 2(-delta delta c(t)) method. Methods.

[32] Euhus D.M., Hudd C., LaRegina M.C., Johnson F.E. (1986). Tumor measurement in the nude mouse. J. Surg. Oncol..

[33] Tomayko M.M., Reynolds C.P. (1989). Determination of subcutaneous tumor size in athymic (nude) mice. Cancer Chemother. Pharmacol..

[34] Zamarin D., Martinez-Sobrido L., Kelly K., Mansour M., Sheng G., Vigil A., Garcia-Sastre A., Palese P., Fong Y. (2009). Enhancement of oncolytic properties of recombinant newcastle disease virus through antagonism of cellular innate immune responses. Mol. Ther..

[35] Zamarin D., Vigil A., Kelly K., Garcia-Sastre A., Fong Y. (2009). Genetically engineered newcastle disease virus for malignant melanoma therapy. Gene Ther..

[36] Zhao H., Janke M., Fournier P., Schirrmacher V. (2008). Recombinant newcastle disease virus expressing human interleukin-2 serves as a potential candidate for tumor therapy. Virus Res..

[37] Bai F., Niu Z., Tian H., Li S., Lv Z., Zhang T., Ren G., Li D. (2014). Genetically-engineered newcastle disease virus expressing interleukin 2 is a potential drug candidate for cancer immunotherapy. Immunol. Lett..

[38] Bian H., Fournier P., Moormann R., Peeters B., Schirrmacher V. (2005). Selective gene transfer *in vitro* to tumor cells via recombinant newcastle disease virus. Cancer Gene Ther..

[39] Shobana R., Samal S.K., Elankumaran S. (2013). Prostate-Specific antigen-retargeted recombinant newcastle disease virus for prostate cancer virotherapy. J. Virol..

[40] Altomonte J., Marozin S., Schmid R.M., Ebert O. (2010). Engineered newcastle disease virus as an improved oncolytic agent against hepatocellular carcinoma. Mol. Ther..

[41] Silberhumer G.R., Brader P., Wong J., Serganova I.S., Gonen M., Gonzalez S.J., Blasberg R., Zamarin D., Fong Y. (2010). Genetically engineered oncolytic newcastle disease virus effectively induces sustained remission of malignant pleural mesothelioma. Mol. Cancer Ther..

[42] Song K.Y., Wong J., Gonzalez L., Sheng G., Zamarin D., Fong Y. (2010). Antitumor efficacy of viral therapy using genetically engineered newcastle disease virus [ndv(f3aa)-gfp] for peritoneally disseminated gastric cancer. J. Mol. Med..

[43] Li P., Chen C.H., Li S., Givi B., Yu Z., Zamarin D., Palese P., Fong Y., Wong R.J. (2011). Therapeutic effects of a fusogenic newcastle disease virus in treating head and neck cancer. Head Neck.

[44] Obuchi M., Fernandez M., Barber G.N. (2003). Development of recombinant vesicular stomatitis viruses that exploit defects in host defense to augment specific oncolytic activity. J. Virol..

[45] Saloura V., Wang L.C., Fridlender Z.G., Sun J., Cheng G., Kapoor V., Sterman D.H., Harty R.N., Okumura A., Barber G.N. (2010). Evaluation of an attenuated vesicular stomatitis virus vector expressing interferon-beta for use in malignant pleural mesothelioma: Heterogeneity in interferon responsiveness defines potential efficacy. Hum. Gene Ther..

[46] Naik S., Nace R., Barber G.N., Russell S.J. (2012). Potent systemic therapy of multiple myeloma utilizing oncolytic vesicular stomatitis virus coding for interferon-beta. Cancer Gene Ther..

[47] Kirn D.H., Wang Y., Le Boeuf F., Bell J., Thorne S.H. (2007). Targeting of interferon-beta to produce a specific, multi-mechanistic oncolytic vaccinia virus. PLoS Med..

[48] Moerdyk-Schauwecker M., Shah N.R., Murphy A.M., Hastie E., Mukherjee P., Grdzelishvili V.Z. (2013). Resistance of pancreatic cancer cells to oncolytic vesicular stomatitis virus: Role of type I interferon signaling. Virology.

